# Integrated Transcriptome Analysis Reveals the Lung miRNA–mRNA Regulatory Network Associated with Avian Pathogenic *E. coli* Infection

**DOI:** 10.3390/vetsci12020095

**Published:** 2025-01-26

**Authors:** Huan Li, Jishuang Tan, Xiaoyi Li, Susan J. Lamont, Hongyan Sun

**Affiliations:** 1 School of Biological and Chemical Engineering, Yangzhou Polytechnic College, Yangzhou 225009, China; 2 College of Animal Science and Technology, Yangzhou University, Yangzhou 225009, China; 3 Department of Animal Science, Iowa State University, Ames, IA 50011, USA

**Keywords:** chicken lungs, avian pathogenic *E. coli*, mRNA-seq, miRNA-seq, inflammatory response

## Abstract

Avian pathogenic *E. coli* (APEC), one of the most common pathogens, can cause localized or systemic infections and lead to significant economic losses in the poultry industry annually. Recently, evidence suggests that microRNAs (miRNAs) play important roles in the host immune response to bacterial infection by targeting mRNAs. However, few studies have examined the immune mechanisms of miRNAs and mRNAs in chicken lungs following APEC infection. Herein, hematoxylin–eosin staining and qRT-PCR were employed to investigate APEC-induced lung inflammation in chickens. RNAseq was used to identify the miRNAs and mRNAs expression profile between the APEC infection group (APEC) vs. the Control group (Control). The results show that APEC can induce lung lesions in chickens and increase the expression levels of inflammatory cytokines (IL1β, IL8, IL6, and TNFα). High-quality sequencing data were obtained, of which more than 93% of the reads can be mapped to the chicken genome. A total of 22 differentially expressed (DE) miRNAs and 608 DE mRNAs were detected in the APEC vs. the Control. Remarkably, 23 regulatory pairs of miRNA–mRNA interactions were identified in chicken lungs upon APEC infection. Further validation revealed that gga-miR-214 could directly target the *RAB37* gene upon APEC infection to modulate the expression of inflammatory cytokine response. This study provides new insights into the host immune response to APEC infection.

## Simple Summary

The economic impact of colibacillosis, a disease caused by avian pathogenic *E. coli* (APEC), is significant for the global poultry industry. The investigation of APEC–host interactions is important for understanding the host in response to APEC infection and developing effective antibacterial strategies. The aim of this study was to determine the transcriptome (mRNAs and miRNAs) regulation in chicken lungs in response to APEC infection. We found 22 and 608 differentially expressed miRNAs and mRNAs, respectively, between APEC-infected and non-infected chickens, in addition to 23 potential miRNA–mRNA interactions. These data will prove invaluable in elucidating the role of miRNA–mRNA interactions in the defense of chicken lungs against APEC.

## 1. Introduction

Avian pathogenic *E. coli* (APEC) is one of the most commonly observed pathogens responsible for extra-intestinal infectious diseases in poultry, including chickens, geese, and ducks [1,2,3]. It can cause local and systemic infections in poultry through various routes, which are collectively referred to as colibacillosis. Colibacillosis can result in weight loss and a reduction in egg production in poultry, and may even lead to carcass necrosis [4,5,6]. The substantial morbidity and mortality caused by colibacillosis results in millions of dollars in economic losses for the poultry industry every year, seriously impeding its development [7,8,9].

At present, colibacillosis is mainly prevented and treated with antimicrobial drugs [10]. However, indiscriminate use of antibiotics has resulted in the emergence of multiple drug resistance in APEC [11,12,13]. Meanwhile, the issue of drug resistance poses a significant challenge to the prevention and control of colibacillosis. Inactivated vaccines for colibacillosis may also be selected for prevention and treatment purposes [14,15]. However, the diversity of APEC serotypes results in a deficiency in cross-protection among different serotypes [14,15]. It is of even greater importance to develop a vaccine that can provide protection against multiple serotypes. It is, therefore, imperative to gain genetic insight into the host immune response and related resistance mechanisms to APEC infection from a genetic perspective.

Studies have shown that miRNAs play a significant role in regulating the immune system by influencing the expression of target mRNAs [16,17,18]. Although mRNA-seq and miRNA-seq can independently identify important biomarkers and provide critical insights into different treatments or infections, these approaches often fail to capture the full complexity of the immune response, which involves interactions across various biological processes. To address this, combining multi-omics data allows for a more thorough exploration of key regulatory elements and intricate networks, providing deeper insights into the molecular mechanisms underlying host responses to bacterial infections.

As APEC can lead to severe respiratory diseases in poultry, the lungs can be employed to identify new candidate regulatory molecules and networks in chickens infected with APEC. In this study, we performed a systematic and in-depth analysis of mRNA-seq and miRNA-seq data from chicken lungs with/without APEC infection to identify crucial miRNA–mRNA interaction networks involved in the host response to APEC infection.

## 2. Materials and Methods

### 2.1. Bacterial Broth Preparation

APEC O78 strain (CVCC1418) was obtained from the Chinese Veterinary Culture Collection Center (CVCC, Beijing, China). The components of lysogeny broth (LB) included tryptone (Yeasen, Shanghai, China) 1 g, yeast extract (Yeasen, Shanghai, China) 0.5 g, NaCl (Yeasen, Shanghai, China) 1 g, Agar (Yeasen, Shanghai, China) 1.5 g, and 100 mL ultrapure water. The bacterial powder was activated and streaked on LB agar plates two days before APEC infection. A single colony was picked and cultured in LB broth medium at 37 °C overnight. The logarithmic phase bacteria were collected for later use.

### 2.2. Animals and APEC Infection

A total of twenty commercial male broilers at 4 weeks of age were challenged with 0.5 mL APEC O78 (10^8^ colony forming units (CFU)) by the intra-air sac inoculation into the left thoracic air sac. Another twenty males were non-challenged but inoculated in the air sac with 0.5 mL phosphate-buffered saline (PBS). The chickens were provided food and water ad libitum. Chickens were euthanized and necropsied after 24 h post-challenge. A veterinary pathologist conducted a visual inspection and assigned scores to lesions in three tissues—air sacs, pericardium, and liver—using the standard pathology scoring system outlined by Peighambari et al. [19]. The clinical picture is displayed in Figure S1. The lungs were harvested, immediately frozen and stored at −80 °C for further analyses.

### 2.3. Cytokine Quantification in Lungs

The concentrations of interleukin 1β (IL1β), interleukin 6 (IL6), tumor necrosis factor α (TNFα), and interleukin 18 (IL18) in the lung were measured separately according to the instructions of the quantikine enzyme-linked immunosorbent assay (ELISA) kit (R&D Systems, Minneapolis, MN, USA).

### 2.4. Histologic Observation in Lungs

A 4% paraformaldehyde solution was used to fix the harvested lung sections. After soaking for 24 h, lung tissues were dehydrated by using a series of ethanol and xylene solutions, and then were embedded in paraffin. Then, hematoxylin–eosin (HE) was used to stain the slices after cutting them into 5-micron-thick pieces using a microtome. A microscope was utilized to observe and evaluate the histomorphology of the lungs.

### 2.5. RNA Isolation and cDNA Library Construction for miRNA and mRNA Sequencing

The RNeasy Kits (QIAGEN, Hilden, Germany) and miRNA Purification Kit-CW0627S (CWBIO, Beijing, China) were used to extract the mRNA and miRNA, respectively, from APEC-infected individuals (APEC, *n* = 3) and non-infected individuals (Control, *n* = 3) according to the manufacturer’s instructions. DNase I was used to digest DNA for the total RNA. A Nanodrop^TM^ OneCspectrophotometer (Thermo Fisher Scientific, Waltham, MA, USA) was used to determine the RNA quality by examining the value of A_260/A280_. Total RNA (2 μg) was used as an input for miRNA library preparation by using the KC Digital^TM^ small RNA Library Prep Kit for Illumina^®^ (Wuhan Seqhealth Co., Ltd., Wuhan, China) following the manufacturer’s instructions. The eluted cDNA library was separated by 6% PAGE gel, after which approximately 160 bp bands were isolated, purified, and quantified by Qseq (Qseq100, Bioptic Inc., Taiwan, China). After miRNA library preparation, samples were finally sequenced on a Hiseq X-10 sequencer (Illumina, San Diego, CA, USA) with a PE150 model. High-quality RNA (2 μg) was performed for mRNA library preparation with the Ribo-off rRNA depletion kit (Illumina, San Diego, CA, USA). Then, mRNA libraries were sequenced on a NovaSeq 6000 sequencer (Illumina, San Diego, CA, USA) with a PE150 sequencing platform.

### 2.6. Analysis of miRNA and mRNA Sequencing Data

Raw miRNA sequencing data were filtered by Fastx_toolkit (version 0.0.13.2) [20] and cutadapt (version 1.15) [21] to remove the adaptor sequences and obtain clean reads. Before mapping, a length filter was performed to keep a small RNA length of 18~35 nt. Subsequently, the clean reads were aligned to the chicken reference genome (*Gallus gallus* 7) by using bowtie2 (version 2.4.3) [22] with default parameters. Reads per million (RPM) were used to normalize the mapped reads, which were then compared to the known abundances of miRNAs in miRBase to obtain the known miRNAs. The mirdeep2 package (version 2.0.0.8) [23] with default parameters was used to predict the novel miRNAs. The expression level of known and new miRNAs in each sample was measured by using the transcripts per million reads (TPM) method. Then, the differentially expressed (DE) miRNAs between the control group (Control) and the APEC infection group (APEC) were determined by the edgeR package (version 3.12.1) [24] with default parameters. The statistical significance of miRNA expression differences was considered as adjusted *p*-value < 0.05 and |fold change| > 2. The miRanda software (version 3.3a) was used to predict the target mRNA of differentially expressed miRNA.

Trimmomatic (version 0.36) [25] with default parameters was used to determine the quality of raw mRNA sequencing data. Then, the STAR software (version 2.5.3a) [26] with default parameters was used to map the clean reads to the chicken reference genome (*Gallus gallus 7*). FeatureCounts (Subread 1.5.1) [27] with default parameters were used to calculate the reads mapped to gene exon regions. The expression level of detected transcript in each sample was determined by using Reads per Kilobase per Million Reads (RPKMs). Then, the edgeR package (version 3.12.1) [24] with default parameters was employed to find the DE genes between the Control and APEC groups. The statistical significance of gene (mRNA) expression differences was determined with the cutoff of adjust *p*-value < 0.05 and |fold change| > 1.5. The overlapped genes, co-expressed DE mRNA–miRNA networks, were found between the target genes of DE miRNAs and genes. Then, the KOBAS software (version 2.1.1) with default parameters was utilized to perform the gene ontology (GO) [28] and KEGG [29,30] enrichment analyses of the overlapped genes with the cut-off of *p* < 0.05 to judge the statistically significant enrichment.

For the integration of miRNA-seq and mRNA-seq data, a Venn diagram was used to explore the overlapped mRNAs between the identified DE mRNAs and the potential target mRNAs of the identified DE miRNA in this study. Then, the overlapped mRNAs and their corresponding miRNAs were the miRNA–mRNA regulation pairs that were used for subsequent analysis.

### 2.7. Vector Construction and Cell Transfection

The 3′UTR of *RAB37* regions with the binding sites of gga-miR-214 were amplified by using PCR technology. Subsequently, the Mut Express II Fast Mutagenesis Kit V2 (Vazyme, Nanjing, China) and the wild type 3′UTR of *RAB37* (template) were used to generate the *RAB37* 3′UTR mutant, which eliminated the predicted binding sites (CTGCTG). The specific primers for the wild type and mutant 3′UTR of *RAB37* are displayed in Table S1. The circular pmirGLO plasmid was cleaved into linear plasmid via restriction enzymes *Xho I* and *Sac I.* Then, the amplified fragments (the wild type and mutant 3′UTR of *RAB37*) were ligated into the linear pmirGLO plasmid by using the homologous recombination method. Finally, Sanger sequencing was used to confirm the successful insertion of the target fragment.

The gga-miR-214 mimics were synthesized by GenePharma (Shanghai, China). In a 24-well plate, each well containing 1 × 10^5^ HD11 macrophages was used for transfection. Subsequently, Lipofectamine™ 8000 reagent (Invitrogen, Carlsbad, CA, USA) was utilized to transfect either the mimic/*RAB37* mutation vector (50 nM) or the negative control (50 nM). Then, the aforementioned transfected cells were challenged with APEC O78 (0.1 mL concentration of 1 × 10^8^ CFU/mL) for 24 h. For the Control group, cells were injected with the same dosage of PBS (0.1 mL) for the same duration. After infection, the cells were harvested for the following experiments.

### 2.8. Dual-Luciferase Reporter Analysis

To validate the interaction between gga-miR-214 and *RAB37*, a dual-luciferase reporter was performed. Lipofectamine 8000™ transfection reagent (Invitrogen, Carlsbad, CA, USA) was utilized to transfect the wild-type/mutant *RAB37* (500 ng) vector and gga-miR-214 mimic (500 ng) into HD11 cells. After 36 h, the Dual-luciferase Reporter Assay Kit (Beyotime, Shanghai, China) was used to identify the luciferase activity.

### 2.9. Quantitative Real-Time PCR (qRT-PCR) Assay

According to the manufacturer’s instructions, total RNA was extracted from lung tissues in different groups. Then, a total of 1 μg RNA for each sample was used to reverse transcribe into cDNA with a reverse transcription kit (Takara, Dalian, China). The primers used in this study are shown in Tables S2 and S3. The One Step SYBR PrimeScript PLUS RTRNA PCR Kit (Takara, Dalian, China) was used for cDNA synthesis. qRT-PCR was conducted using a SYBR Premix Ex Taq II kit (Takara, Dalian, China) to measure the expression level of candidate genes. qRT-PCR thermal cycling conditions were as follows: denaturation at 95 °C for 3 min, 40 cycles at 95 °C for 10 s, at 58 °C for 30 s, and then at 72 °C for 30 s. The 2^−△△Ct^ method was used to calculate the relative expression of the candidate gene [31].

### 2.10. Statistical Analyses

Statistical analysis was carried out by using paired *t*-tests with JMP statistical software (version 15.2.1, SAS Institute, Cary, NC, USA). Data were expressed as the mean ± standard deviation. Statistical significance was defined at *p* < 0.05.

## 3. Results

### 3.1. APEC-Induced Lung Inflammation and Damage in Chicken

**Figure 1:**
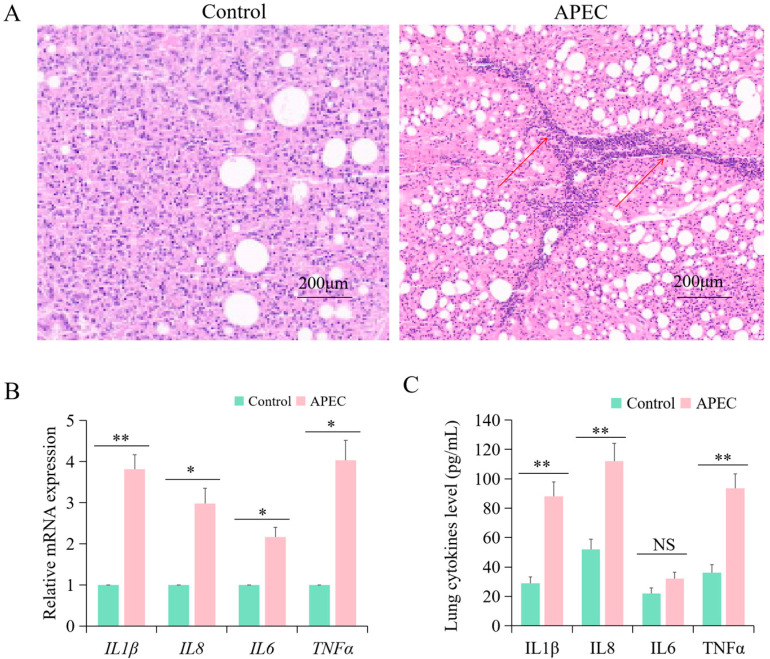
APEC-induced lung inflammation and damage at 24 h in chicken. (**A**) Lung slices were stained with hematoxylin–eosin to assess tissue injury. The red arrow indicates that eosinophilic material and white blood cells were observed in many vascular and parabronchial lumens. (**B**) The mRNA abundance of *IL1β*, *IL8*, *IL6*, and *TNFα* assessed in the lungs by qRT-PCR. * *p* < 0.05; ** *p* < 0.01. (**C**) The concentrations of IL1β, IL8, IL6, and TNFα in the lungs were measured by ELISA. * *p* < 0.05; ** *p* < 0.01; NS, not significant.

HE staining showed that control lungs exhibited complete histological structures. In contrast, lungs from the APEC group exhibited damaged structures, enlarged intercellular space, increased eosinophilic material, and white blood cells (Figure 1A). These morphological results suggested that APEC infection can induce lung lesions in chickens. The concentrations of inflammatory cytokines were measured to determine the effects of APEC. As shown in Figure 1B, the mRNA expression levels of *IL1β*, *IL6*, *IL8,* and *TNFα* in the lungs of the APEC group were significantly increased than those in the Control group. Meanwhile, the concentrations of IL1β, IL8, and TNFα were also significantly higher in the APEC group in comparison to the Control group (Figure 1C). There was no discernible difference between the concentration of IL6 in the APEC group and that in the Control group (Figure 1C).

### 3.2. Overview of the High-Throughput Sequencing Data for Chicken Lung Tissues with APEC Infection

A total of six miRNA libraries were established from the wild-type lung (Control, n = 3) and APEC infection group (APEC, n = 3), which were high-throughput sequenced using the Illumina Hiseq X-10 platform (Seqhealth, Wuhan, China) analysis. In total, 43,479,222~49,902,970 raw reads were generated (Table 1), of which 43,369,696~49,774,208 clean reads were obtained (Table 1). Moreover, a unique identifier (UID) was also used to remove the duplication data, resulting in a total of 34,854,086~44,373,224 UID reads (Table 1). The average GC content of the clean reads was 45.77%. To further evaluate the changes of small RNAs in the lung during APEC infection, the length distribution of all small RNA reads in the nine libraries was analyzed. The majority of the unique small RNAs from the nine libraries ranged from 18 to 24 nt, of which 22 nt in length was the predominant read, followed by 21, 23, and 20 nt in length (Figure S2). The length distribution of small RNA in the current study was consistent with the typical sizes of dicer processing products. The clean reads were then aligned to the chicken reference genome (*gallus gallus* 7) and Rfam to annotate the categories of the non-coding RNAs. The results showed that an average of 2,369,042 (94.19%) and 2,166,524 (93.94%) of total clean small RNA reads were mapped to the chicken genome, which was obtained from the control and APEC group, respectively (Table 2). The reads of each sample that were mapped into the intergenic, intron, promoter, exon, 3′ untranslated regions (3′UTR), and 5′ untranslated regions (5′UTR) are displayed in Table 3. A total of six mRNA libraries with three biological replicates of two groups (Control and APEC) were successfully established. It was found that from 90,658,838 to 108,900,922 raw reads were detected from the six individuals, which generated from 76,540,890 to 92,315,076 clean reads after quality control (Table 4). Moreover, a total of 54,137,065~60,859,692 can be successfully mapped to the chicken reference genome (Table 4).

### 3.3. Known and Novel miRNA Analysis

**Figure 2:**
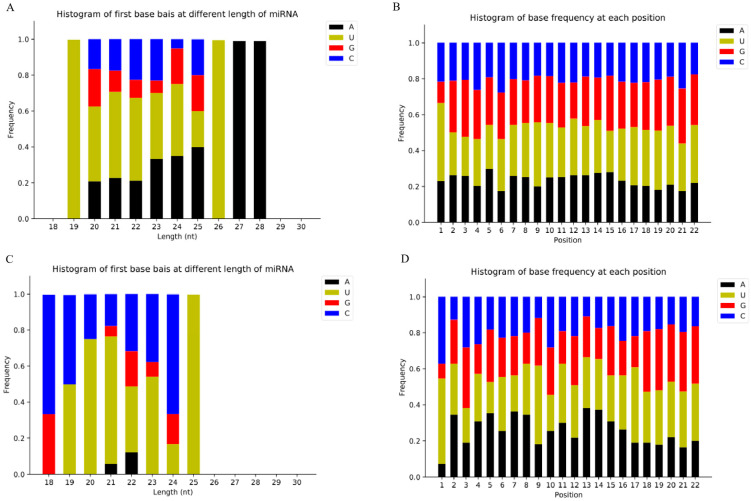
First miRNA nucleotide bias analysis results. (**A**,**C**). Proportion of known (**A**) and novel miRNA (**C**) first nucleotide bias. (**B**,**D**). Proportion of known (**B**) and novel miRNA (**D**) nucleotide bias at each position.

After comparing the mapped reads to the specific range of sequence in miRBase, it was found that the APEC group contained an average of 339 known hairpins and 446 mature miRNAs, while the Control group contained an average of 351 known hairpins and 458 mature miRNAs (Table S4). A summary of the first nucleotide bias and its nucleotide bias at each position of the known miRNA is shown in Figure 2A,B. Among the six samples of the Control and APEC group, the first nucleotide base analysis showed that uridine (U) was the strongest preference in 27- and 28-nucleotide long miRNA (Figure 2A). The first base of adenine (A) was the strongest preference for 19 and 26 nucleotides. The analysis of nucleotide bias at each position of the known miRNA showed that the miRNAs had the major preference for U at position 1. The base preferences were roughly equivalent at other positions (Figure 2B). A total of 13,639 potential novel miRNAs were found to map into the chicken genome, of which an average of 113 potential novel mature miRNAs were identified. The first base of U was the strongest bias for 25 nucleotides (Figure 2C). Generally, the base bias at each position, except position 1 (predominant with base U), was performed approximately equally in each of those detected novel miRNAs (Figure 2D).

### 3.4. Identification of Differentially Expressed (DE) miRNAs and mRNAs

**Figure 3:**
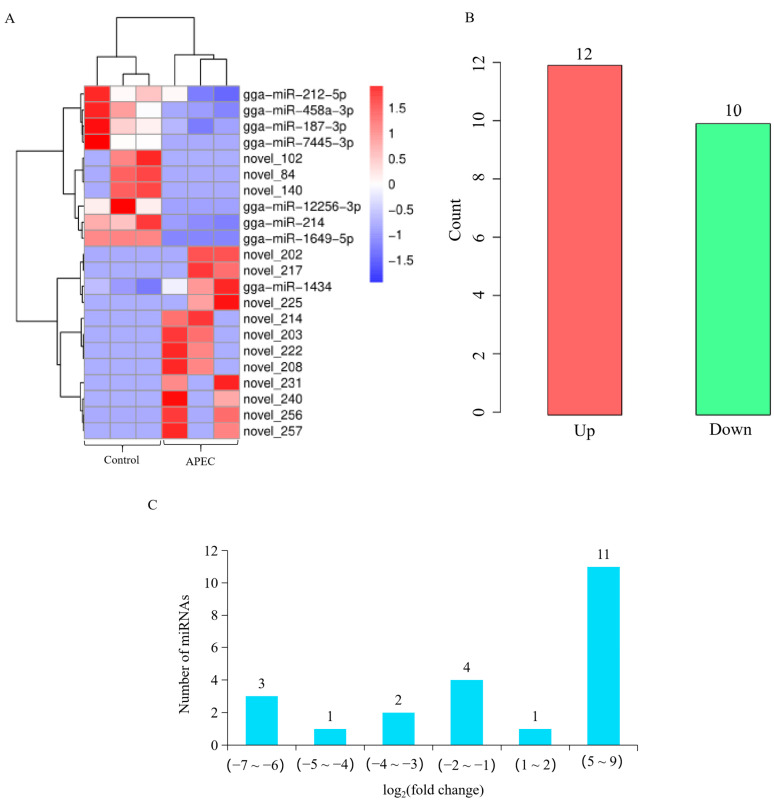
The miRNA-seq profiling in the comparisons of the APEC vs. Control. (**A**) Heatmap analysis for the miRNA-seq data from the comparison of APEC vs. Control. The red color indicated up-regulated miRNAs, while the blue color represented down-regulated miRNAs. (**B**) Numbers of differentially expressed miRNAs in the APEC vs. Control. (**C**) The distribution of the differentially expressed miRNAs in the comparison of the APEC vs. Control.

**Figure 4:**
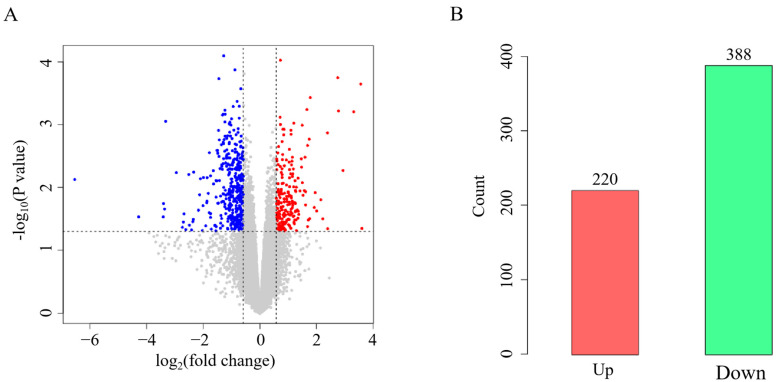
The mRNA-seq profiling in the comparisons of the APEC vs. Control. (**A**) Volcano plot for the differentially expressed mRNAs in the comparison of the APEC vs. Control. The red color indicated up-regulated mRNAs, while the blue color represented down-regulated mRNAs. (**B**) Numbers of differentially expressed mRNAs in the APEC vs. Control. The red color indicated up-regulated mRNAs, while the green color represented down-regulated mRNAs.

To screen the differential expression profiles of miRNA in chicken lungs with or without APEC infection, a cluster analysis of the DE miRNAs was displayed in Figure 3A. The miRNA expression pattern of the APEC group was completely different from that of the Control group (Figure 3A). In general, a total of 22 (12 up-regulated and 10 down-regulated) DE miRNAs were identified in the comparison of the APEC vs. Control (Figure 3B and Table S5). Among the 22 DE miRNAs, there were 8 (7 down-regulated and 1 up-regulated) known miRNAs and 14 novel miRNAs (3 down-regulated and 11 up-regulated) in the APEC vs. Control. It was noteworthy that 9 out of the 10 up-regulated DE miRNAs displayed a log_2_ (fold change) value greater than 5 (Figure 3C). Compared with the control group, a total of 608 DE mRNAs were identified in the APEC group, including 220 up-regulated and 388 down-regulated DE mRNAs (Figure 4A,B and Table S6).

### 3.5. Construction of the miRNA–mRNA Regulation Pairs

Based on the miRNA regulatory mechanism, we integrated the DE mRNAs and the target mRNAs of the DE miRNAs to identify the miRNA–mRNA regulation pairs. A total of 23 miRNA–mRNA interactions were found in the comparison of the APEC vs. Control (Table 4). Among the miRNA–mRNA interactions, gga-miR-1649-5p had 11 target genes, followed by gga-miR-214 with 6 targets, gga-miR-12256-3p with 4 targets, and gga-miR-212-5p with 2 targets (Table 5).

### 3.6. Identification of the miRNA–mRNA Interactions Under APEC Infection

**Figure 5:**
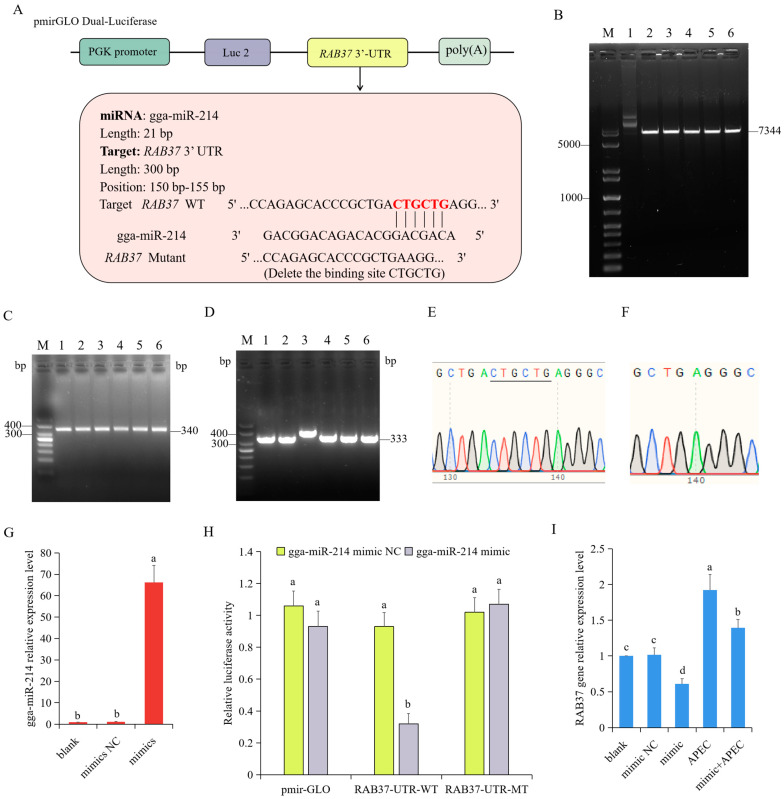
Identification of the gga-miR-214 and *RAB37* interaction. (**A**) The information of gga-miR-214 and *RAB37* interaction and their binding sites. (**B**) The pmirGLO plasmid was digested by restriction enzyme *Xho I* and *Sac I*. M, marker; 1, pmirGLO plasmid; 2–6, plasmid digested by *Xho I* and *Sac I*. (**C**) Construction of the wild type *RAB37* 3′UTR. M, Marker; 1–6, The PCR product amplified by the wild type *RAB37* 3′UTR primers. (**D**) Construction of the mutant of *RAB37* 3′UTR. M, Marker; 1–6, The PCR product amplified by the mutant *RAB37* 3′UTR primers using the wild type *RAB37* 3′UTR plasmid as a template. (**E**,**F**) The sequencing result of wild-type (**E**) and mutant (**F**) 3′UTR of *RAB37*. (**G**) The expression level of gga-miR-214 was detected after chicken macrophages were transfected with a gga-miR-214 mimic. (**H**) A dual-luciferase reporter assay was performed to investigate the relationship between gga-miR-214 and *RAB37*. (**I**) The expression level of *RAB37* was detected after chicken macrophages were transfected with a gga-miR-214 mimic. Different letters indicated *p* < 0.05. The same letters represented *p* > 0.05.

To validate the reliability of the identified miRNA–mRNA interactions (gga-miR-214 and *RAB37*) during APEC infection (Figure 5A), a dual-luciferase reporter assay was performed. Firstly, we used the restriction enzymes *Xho I* and *Sac I* to digest the pmirGLO luciferase plasmid (Figure 5B). Meanwhile, a wild type (pmirGLO-RAB37-WT) or mutant (pmirGLO-RAB37-MT) 3′UTR of *RAB37* was constructed (Figure 5C,D). The sequencing results revealed that the recombinant plasmids pmirGLO-RAB37-WT and pmirGLO-RAB37-MT were successfully constructed (Figure 5E,F and Figures S3 and S4). Then, the RT-qPCR results showed that the expression of gga-miR-214 was significantly increased after chicken macrophages were transfected with a gga-miR-214 mimic (*p* < 0.05, Figure 5G). Finally, a dual-luciferase reporter assay was conducted after chicken macrophages were co-transfected with pmirGLO-RAB37-WT or pmirGLO-RAB37-MT and either gga-miR-214 mimic for 36 h. It was found that the gga-miR-214 mimic can significantly decrease the luciferase activity from the wild-type *RAB37* 3′UTR reporter plasmid (*p* < 0.05, Figure 5H). Furthermore, the gga-miR-214 mimic can significantly reduce the expression level of *RAB37* with or without APEC infection (*p* < 0.05, Figure 5I). These findings revealed that *RAB37* is the target gene of gga-miR-214 upon APEC infection.

### 3.7. qRT-PCR Validation for the High-Throughput Data

**Figure 6:**
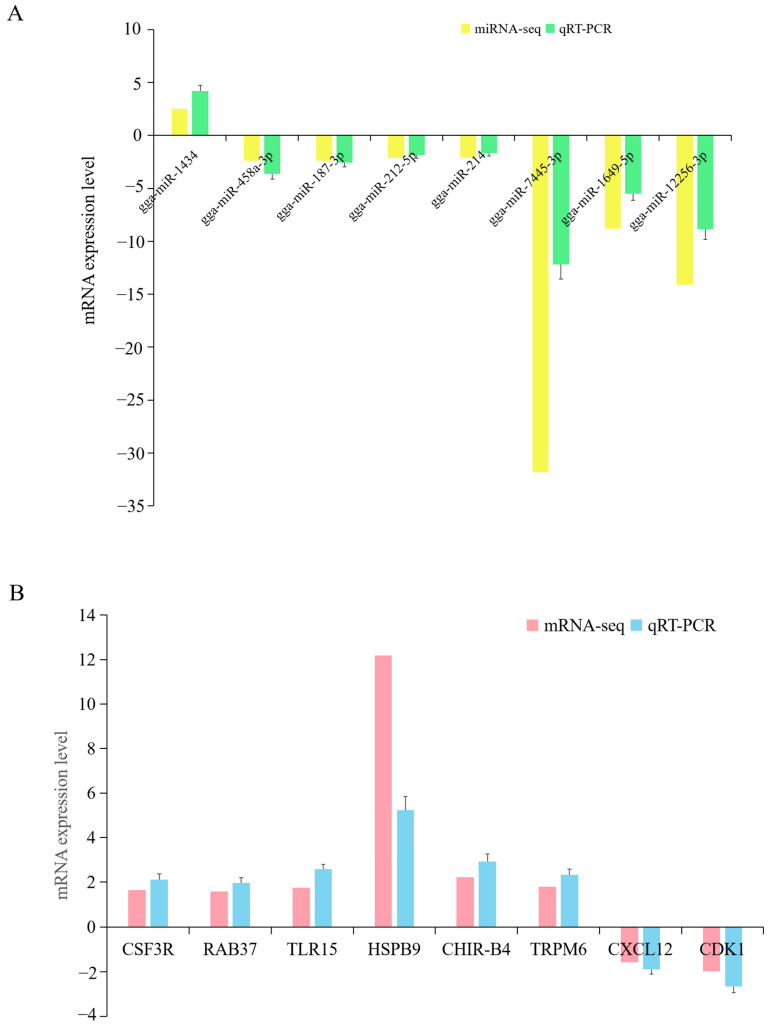
Verification of the high-throughput sequencing data by qRT-PCR for the comparison of the APEC infection group vs. Control group. A-B. Verification of miRNA-seq data (**A**) and mRNA-seq (**B**) by qRT-PCR for the comparison of the APEC infection group vs. Control group.

To validate the reliability of the high-throughput data, a qRT-PCR was used to measure the expression level of selected DE miRNAs and mRNAs in the comparison of the APEC vs. Control, including eight DE miRNAs (gga-miR-1434, gga-miR-458a-3p, gga-miR-187-3p, gga-miR-212-5p, gga-miR-214, gga-miR-1649-5p, gga-miR-12256-3p, and gga-miR-7445-3p) and eight DE mRNAs (*CSF3R*, *RAB37*, *TLR15*, *HSPB9*, *CHIR-B4*, *TRPM6*, *CXCL12*, *CDK1*). The results showed that the changes in the direction of the DE miRNAs and mRNAs expression levels in qRT-PCR were in agreement with the high-throughput data (Figure 6), indicating the high reliability of the expression profile.

## 4. Discussion

Currently, respiratory diseases such as avian colibacillosis have caused significant losses to the poultry industry [32,33]. The lungs are the primary target organ of many bacterial diseases in poultry. For example, APEC initially infiltrates chicken through the mucosal surface of the pulmonary respiratory system and then spreads to other organs [34]. Therefore, the health of animals largely depends on whether they can successfully control the invasion and replication of pathogens in lung tissues, especially in the bronchial mucosa. To study the pathogenesis of avian colibacillosis and take effective prevention and control measures, it is necessary to understand the function of the lung-related immune system. Since single mRNA-seq and miRNA-seq can explore the important mRNAs and miRNAs, respectively, involved in bacterial infection [35,36], integration of the mRNA-seq and miRNA-seq data can comprehensively provide more information on the regulation network and reveal the underlying immune response.

In the present study, it was found that pathological changes related to inflammation in the lungs with APEC infection, such as cell swelling and laminar inflammatory proliferation. The expression levels of cytokines were also extensively up-regulated upon APEC infection. These results indicate that the lung is a target organ during APEC infection and APEC can cause inflammation and injury in chicken lungs. Previous research has shown similar results [37,38]. APEC can significantly increase the expression level of inflammatory cytokines. For the miRNA-seq and mRNA-seq experiments, more than 93% of reads can be successfully mapped to the chicken genome, indicating that the majority of sequencing data can be effectively located on the genome. These high-quality data ensure the accurate subsequent analysis of gene expression, which is helpful in understanding the biological processes against APEC infection. According to the integration of the multi-omics data, we identified 23 pairs of potential miRNA–mRNA interactions in the comparison of the APEC vs. Control. Moreover, we demonstrated that there is a real regulatory relationship between gga-miR-214 and *RAB37* under APEC infection. Both gga-miR-214 and *RAB37* played diverse important roles in immune response.

It was found that gga-miR-214 was able to participate in chicken immune response [39] and skeletal muscle development [40]. Remarkably, Chu et al. demonstrated that gga-miR-214 was able to inhibit the NFκB-mediated bacteria-triggered inflammatory response by targeting the *myd88* gene in fish [41]. In the current study, the expression of gga-miR-214 was significantly down-regulated (fold change = −2.11) in chicken lungs after APEC infection, indicating the expression of gga-miR-214 was inhibited in APEC-triggered inflammatory response. However, the expression of *RAB37* was significantly up-regulated (fold change = 1.66) in the lungs of the same individuals after APEC infection, which is negatively correlated with gga-miR-214.

According to previous findings in the literature, the results observed in our study are reasonable. Kuo et al. have demonstrated that *RAB37* can positively regulate the secretion of IL-6 and link with IL-6/STAT3/PD-1 transcription regulation in macrophages to foster an immunosuppressive tumor microenvironment [42]. Meanwhile, Wang et al. found that the down-regulation of *RAB37* can inhibit inflammation and autophagy in ventilator-induced lung injury in mice [43]. Consistent with these findings, we observed that *RAB37* was the direct target gene of gga-miR-214 upon APEC infection to modulate the expression of the inflammatory cytokine response. In the future, we can modulate the expression level of gga-miR-214 to further regulate the excessive expression of inflammation-related genes, avoiding serious damage to the host caused by inflammatory storms. Meanwhile, the gga-miR-214 mimic can be used to enhance the antimicrobial capacity of the host.

## 5. Conclusions

In conclusion, we identified and characterized the miRNA and mRNA expression profiles in chicken lungs before and after APEC infection. A total of 22 DE miRNAs and 608 DE genes were detected in the comparison of the APEC vs. Control. In addition, 23 pairs of miRNA–mRNA interactions were identified to participate in the immune response upon APEC infection. Furthermore, *RAB37* was the direct target gene of gga-miR-214 upon APEC infection to modulate the expression of inflammatory cytokine response. This study characterized the miRNA and mRNA expression and provided new insights into the host’s immune response against APEC infection.

## Figures and Tables

**Table 1:** Summary of high-throughput miRNA sequencing data.

Groups	Raw Reads	Clean Reads	Reads with UIDs	Deduplicate Reads
Control_1	46,188,862	46,078,160	39,986,032 (86.78%)	8,523,900 (18.50%)
Control_2	46,857,762	46,724,126	42,694,530 (91.38%)	8,550,132 (18.30%)
Control_3	46,427,898	46,312,776	42,684,074 (92.16%)	8,657,362 (18.69%)
APEC_1	49,902,970	49,774,208	44,373,224 (89.15%)	7,939,504 (15.95%)
APEC_2	44,639,162	44,508,316	39,748,056 (89.30%)	8,205,638 (18.44%)
APEC_3	43,479,222	43,369,696	34,854,086 (80.37%)	8,240,754 (19.00%)

Note: The “clean reads” indicated the reads after quality control (removing low-quality bases, sequencing adapters, etc.).

**Table 2:** The miRNA-seq reads mapping information for each group.

Reads	Control_1	Control_2	Control_3	APEC_1	APEC_2	APEC_3
Clean total reads	2,404,069	2,571,083	2,570,090	2,323,328	2,412,368	2,182,006
Clean unique reads	433,612	489,753	531,798	426,729	441,966	372,738
Mapped total reads	2,256,838	2,424,036	2,426,251	2,187,424	2,271,269	2,040,879
Mapped unique reads	391,669	443,789	483,368	382,585	398,407	331,739
Total mapping rate	93.88%	94.28%	94.4%	94.15%	94.15%	93.53%
Unique mapping rate	90.33%	90.61%	90.89%	89.66%	90.14%	89.00%

Note: The “clean total reads” represented the total reads after the length filter (only keeping a small RNA length of 18~35 nt).

**Table 3:** Distribution of miRNAs mapping reads on the chicken genome.

Mapped Region Types	Control_1	Control_2	Control_3	APEC_1	APEC_2	APEC_3
Promoter	565,030	594,927	596,361	594,287	600,101	584,033
5′UTR	57,616	59,133	62,722	52,413	52,704	52,006
Exon	162,857	173,472	180,611	133,605	157,659	119,356
Intron	597,966	631,074	656,954	555,887	576,698	535,047
3′UTR	136,543	152,700	166,529	125,689	132,972	113,511
Intergenic	6,395,694	6,670,854	6,734,144	5,215,841	5,621,161	5,490,755

**Table 4:** Summary of high-throughput mRNA sequencing data.

Groups	Raw Reads	Clean Reads	UID Reads	Total Mapped Reads	Unique Mapped Reads
Control_1	108,900,922	92,315,076	65,356,410	60,859,692 (93.12%)	58,266,983 (95.74%)
Control_2	95,851,924	81,885,132	62,389,512	58,057,547 (93.06%)	56,020,822 (96.49%)
Control_3	90,658,838	76,540,890	58,025,610	54,137,065 (93.30%)	52,164,176 (96.36%)
APEC_1	92,958,930	78,999,574	60,753,500	56,250,074 (92.59%)	54,033,269 (96.06%)
APEC_2	92,886,340	78,420,516	58,481,020	54,373,787 (92.98%)	52,247,961 (96.09%)
APEC_3	95,451,360	80,257,402	61,511,190	57,189,896 (92.97%)	54,934,499 (96.06%)

**Table 5:** The identified miRNA–mRNA regulation pairs upon APEC infection.

miRNA	Length of miRNA (nt)	Gene	Official Full Name
gga-miR-214	21	*RAB37*	member RAS oncogene family
gga-miR-214	21	*IL1RAPL1*	interleukin 1 receptor accessory protein like 1
gga-miR-214	21	*KIF4B*	kinesin family member 4B
gga-miR-214	21	*LOC100857964*	uncharacterized
gga-miR-214	21	*AvBD1*	avian beta-defensin 1
gga-miR-214	21	*SCN4B*	sodium voltage-gated channel beta subunit 4
gga-miR-1649-5p	20	*ARL10*	ADP ribosylation factor like GTPase 10
gga-miR-1649-5p	20	*CSF3R*	colony stimulating factor 3 receptor
gga-miR-1649-5p	20	*FN1*	fibronectin 1
gga-miR-1649-5p	20	*LOC101749531*	uncharacterized
gga-miR-1649-5p	20	*LOC107057545*	uncharacterized
gga-miR-1649-5p	20	*LOC121113126*	basic proline-rich protein-like
gga-miR-1649-5p	20	*N6AMT1*	N-6 adenine-specific DNA methyltransferase 1
gga-miR-1649-5p	20	*PRC1*	protein regulator of cytokinesis 1
gga-miR-1649-5p	20	*SLAMF1*	signaling lymphocytic activation molecule family member 1
gga-miR-1649-5p	20	*ST6GALNAC6*	ST6 N-acetylgalactosaminide alpha-2,6-sialyltransferase 6
gga-miR-1649-5p	20	*TRAIP*	TRAF interacting protein
gga-miR-12256-3p	22	*CHIR-B4*	cluster homolog of immunoglobulin like receptor 4B 2
gga-miR-12256-3p	22	*CLSPN*	claspin
gga-miR-12256-3p	22	*HSPB9*	heat shock protein family B (small) member 9
gga-miR-12256-3p	22	*SHISA4*	shisa family member 4
gga-miR-212-5p	23	*C2orf40*	C2orf40 homolog (augurin)
gga-miR-212-5p	23	*TRPM6*	transient receptor potential cation channel subfamily M member 6

## Data Availability

The data will be available from the corresponding author upon request.

## Supplementary Materials

The following supporting information can be downloaded at https://www.mdpi.com/article/10.3390/vetsci12020095/s1: Figure S1: The clinical picture of liver (A) and heart (B) with or without 24h APEC infection; Figure S2: The percentages of each length of miRNAs for each sample; Figure S3: The whole sequences of the *RAB37* 3’UTR mutant recombinant plasmids; Figure S4: The whole sequences of the *RAB37* 3’UTR wild type recombinant plasmids; Table S1: The specific primers for the wild type and mutant 3′UTR of *RAB37*; Table S2: Primers for candidate miRNAs by qRT-PCR; Table S3: Primers for candidate genes; Table S4: The known hairpin and mature miRNAs in different group; Table S5: The differentially expressed miRNAs between APEC and control group; Table S6: The differentially expressed mRNAs between APEC and control group.
