# Supplementary material for: Integrated Transcriptome Analysis Reveals the Lung miRNA–mRNA Regulatory Network Associated with Avian Pathogenic E. coli Infection
Source: Vet Sci. 2025 Jan 26;12(2):95. doi: 10.3390/vetsci12020095 (PMC11860573; doi:10.3390/vetsci12020095)
Supplement: Supplementary file 1 [file vetsci-12-00095-s001.zip › vetsci-3307142-supplementary/supplementary file/supplementary tables/Table S1.docx]

Table S1 The specific primers for the wild type and mutant 3′UTR of *RAB37*

| **Name** | **Forward (5′-3′)** | **Reverse (5′-3′)** |
| --- | --- | --- |
| wild type 3′UTR of *RAB37* | tctagttgtttaaacgagctCTGCACAGGCCCTGATTC | cctgcaggtcgactctagacGATGGGTTTGTCTGGAAGG |
| mutant 3′UTR of *RAB37* | CTGAGGGCACTTCCAGTACACGCAGCACTTTGG | TACTGGAAGTGCCCTCAGCGGGTGCTCTGGGGG |
