# Supplementary material for: Integrated Transcriptome Analysis Reveals the Lung miRNA–mRNA Regulatory Network Associated with Avian Pathogenic E. coli Infection
Source: Vet Sci. 2025 Jan 26;12(2):95. doi: 10.3390/vetsci12020095 (PMC11860573; doi:10.3390/vetsci12020095)
Supplement: Supplementary file 1 [file vetsci-12-00095-s001.zip › vetsci-3307142-supplementary/supplementary file/supplementary tables/Table S2.docx]

Table S2 Primers for candidate miRNAs by qRT-PCR

| Name | Primer sequence(5’-3’) |
| --- | --- |
| gga-miR-1434 | GUGCGUGAUGAUGGAAAAUU |
| gga-miR-458a-3p | AUAGCUCUUUGAAUGGUACUGC |
| gga-miR-187-3p | UCGUGUCUUGUGUUGCAGCC |
| gga-miR-212-5p | ACCUUGGCUCUAGACUGCUUACU |
| gga-miR-214 | ACAGCAGGCACAGACAGGCAG |
| gga-miR-1649-5p | UCCUGCAGAAGGUGCGGCUG |
| gga-miR-12256-3p | UCUCACUGCUCCUCUUUCCUCA |
| gga-miR-7445-3p | GGAUGAGUCUGUUUAACUUUAU |
