# Supplementary material for: Integrated Transcriptome Analysis Reveals the Lung miRNA–mRNA Regulatory Network Associated with Avian Pathogenic E. coli Infection
Source: Vet Sci. 2025 Jan 26;12(2):95. doi: 10.3390/vetsci12020095 (PMC11860573; doi:10.3390/vetsci12020095)
Supplement: Supplementary file 1 [file vetsci-12-00095-s001.zip › vetsci-3307142-supplementary/supplementary file/supplementary tables/Table S3.docx]

Table S3 Primers for candidate genes

| **Name** | **Forward (5′-3′)** | **Reverse (5′-3′)** |
| --- | --- | --- |
| *CSF3R* | GTGGAACGGCACCAAGCA | GCACGTCAGCCCATAGTCA |
| *β-actin* | CAGCCAGCCATGGATGATGA | ACCAACCATCACACCCTGAT |
| *IL1β* | GCCGAGGAGCAGGGACTTT | ACTGTGAGCGGGTGTAGCG |
| *IL8* | GAGTTCACTGACCACCCT | TGCCTGAGCCATACCTTT |
| *IL6* | TTATGGAGAAGACCGTGAG | GTGGCAGATTGGTAACAGA |
| *TNFα* | CGTTCGGGAGTGGGCTTTA | TTGTGGGACAGGGTAGGG |
| *RAB37* | TCCTGCTCTACGACATCACC | GCTCACGTCGGCCTTATT |
| *TLR15* | CACTGGAAACAGGAGGAT | AAATGGGAAGGAAGACAC |
| *HSPB9* | ACAACGCTCCCAACTCCC | AGGTGCATCCGGCAAAGC |
| *CHIR-B4* | TAATGTGACCCTGGAAGTG | GGGCAACAAGGAGGAAAG |
| *TRPM6* | ATGATGGGACAATAGGCA | CCACCACTAACCCAACTA |
| *U6* | CAAGGACCCATCGTTCCACA | CCATTGGACACGCAGAATGC |
