# Supplementary figures and images for: Integrated Transcriptome Analysis Reveals the Lung miRNA–mRNA Regulatory Network Associated with Avian Pathogenic E. coli Infection
Source: Vet Sci. 2025 Jan 26;12(2):95. doi: 10.3390/vetsci12020095 (PMC11860573; doi:10.3390/vetsci12020095)

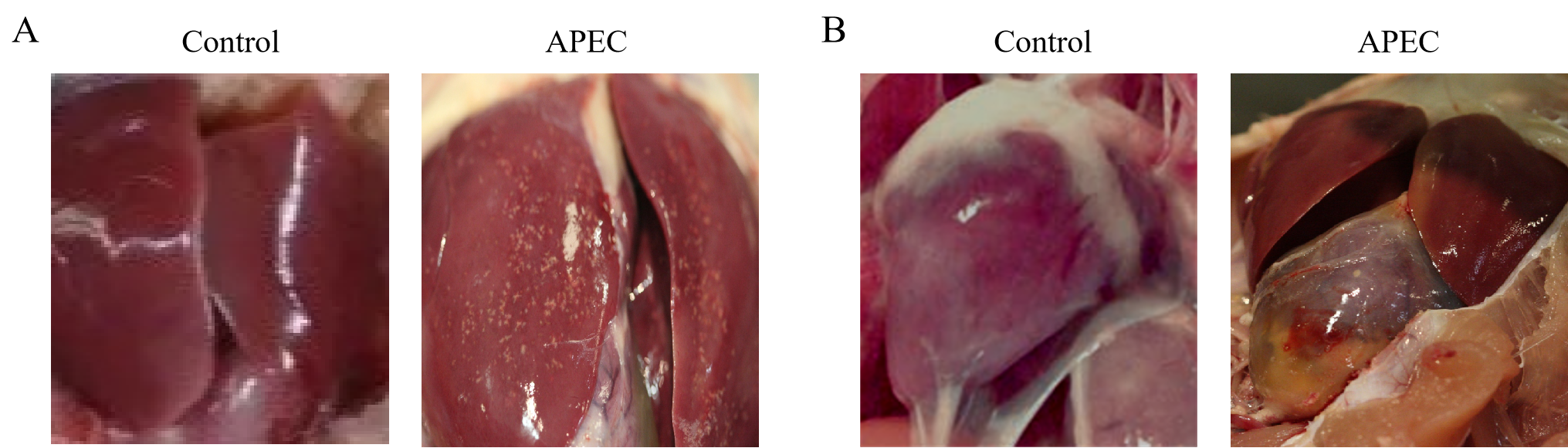

Supplement: Supplementary file 1 [file vetsci-12-00095-s001.zip › vetsci-3307142-supplementary/supplementary file/supplementary figures/Figure S1 clinical picture.png]

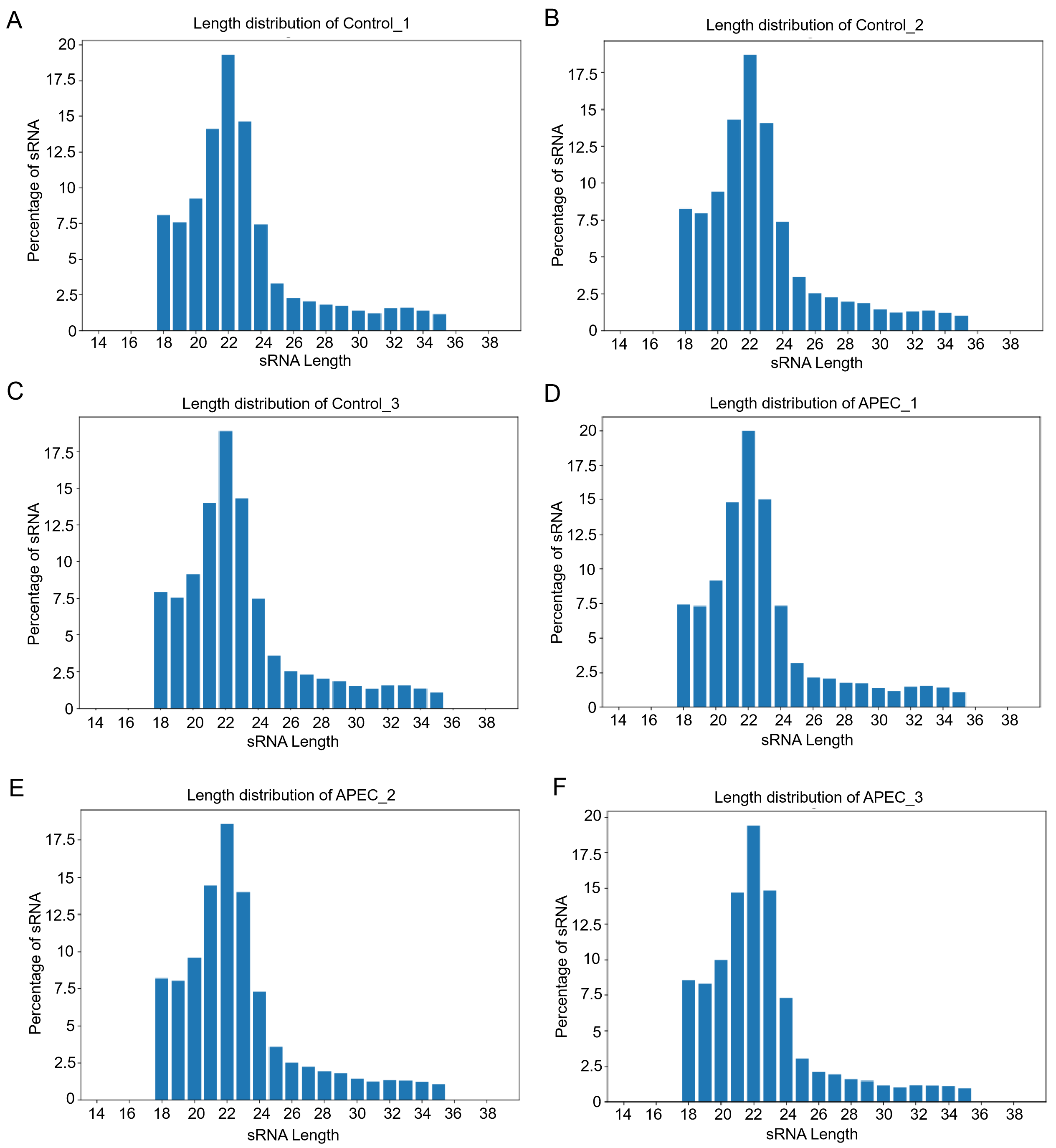

Supplement: Supplementary file 1 [file vetsci-12-00095-s001.zip › vetsci-3307142-supplementary/supplementary file/supplementary figures/Figure S2.png]

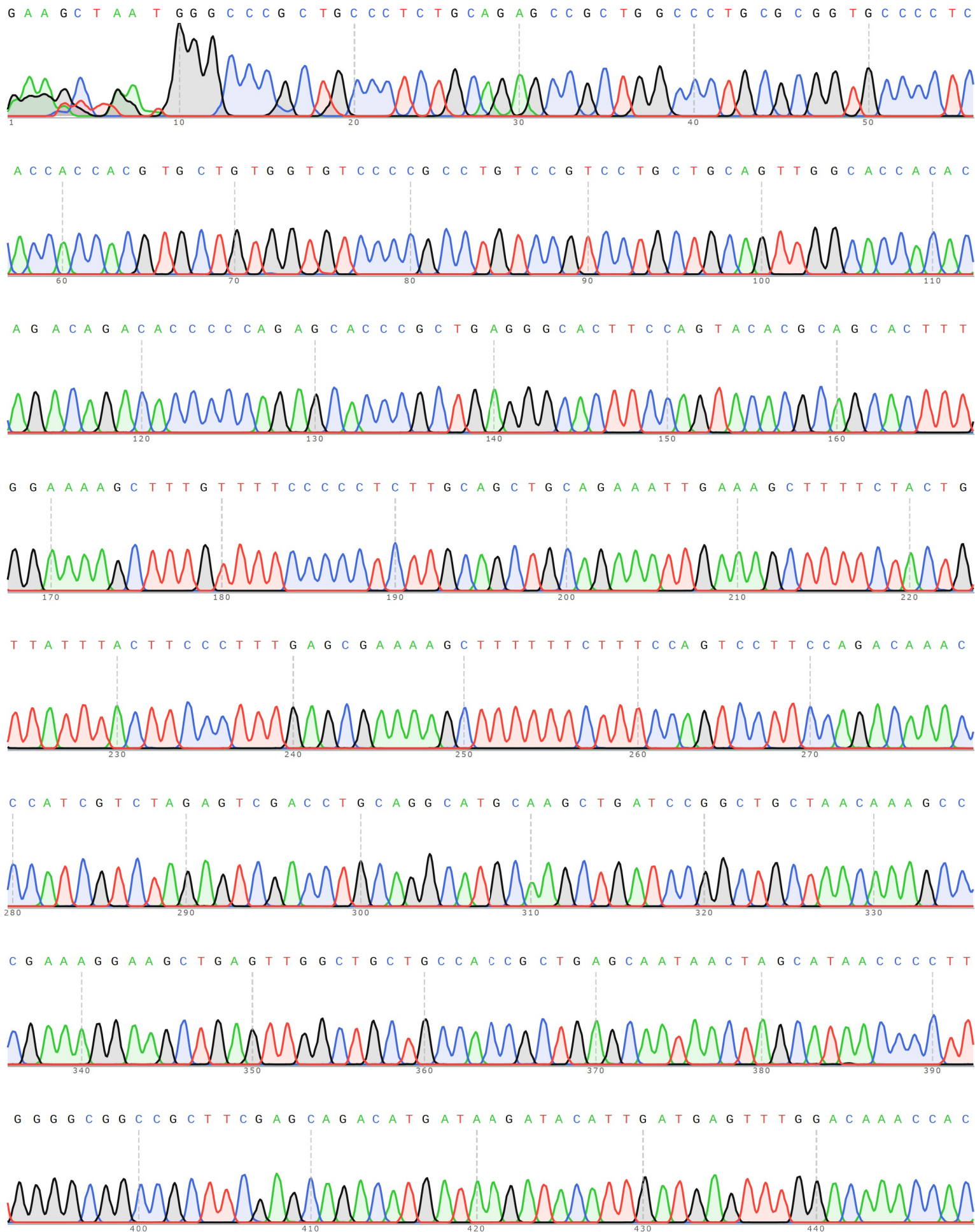

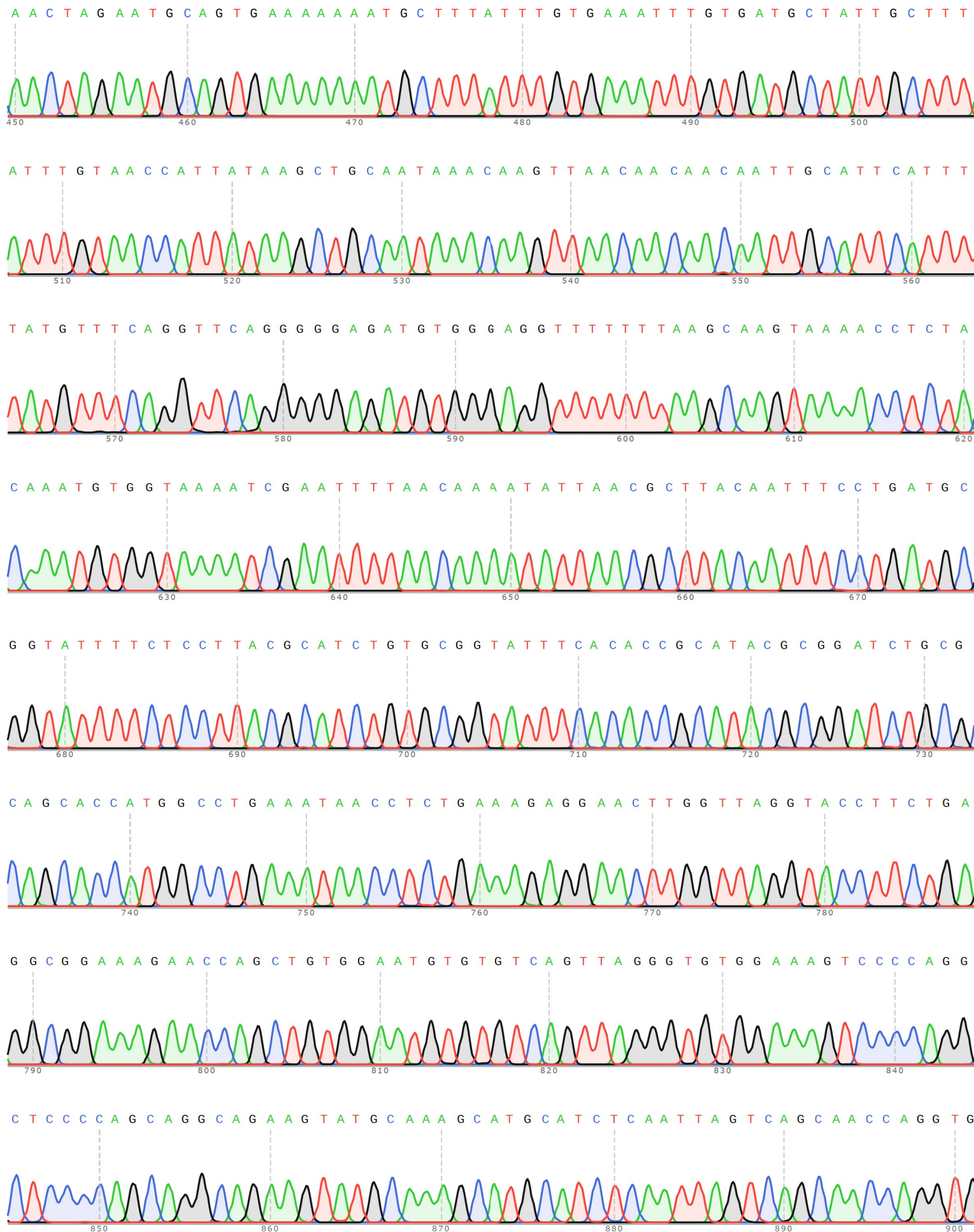

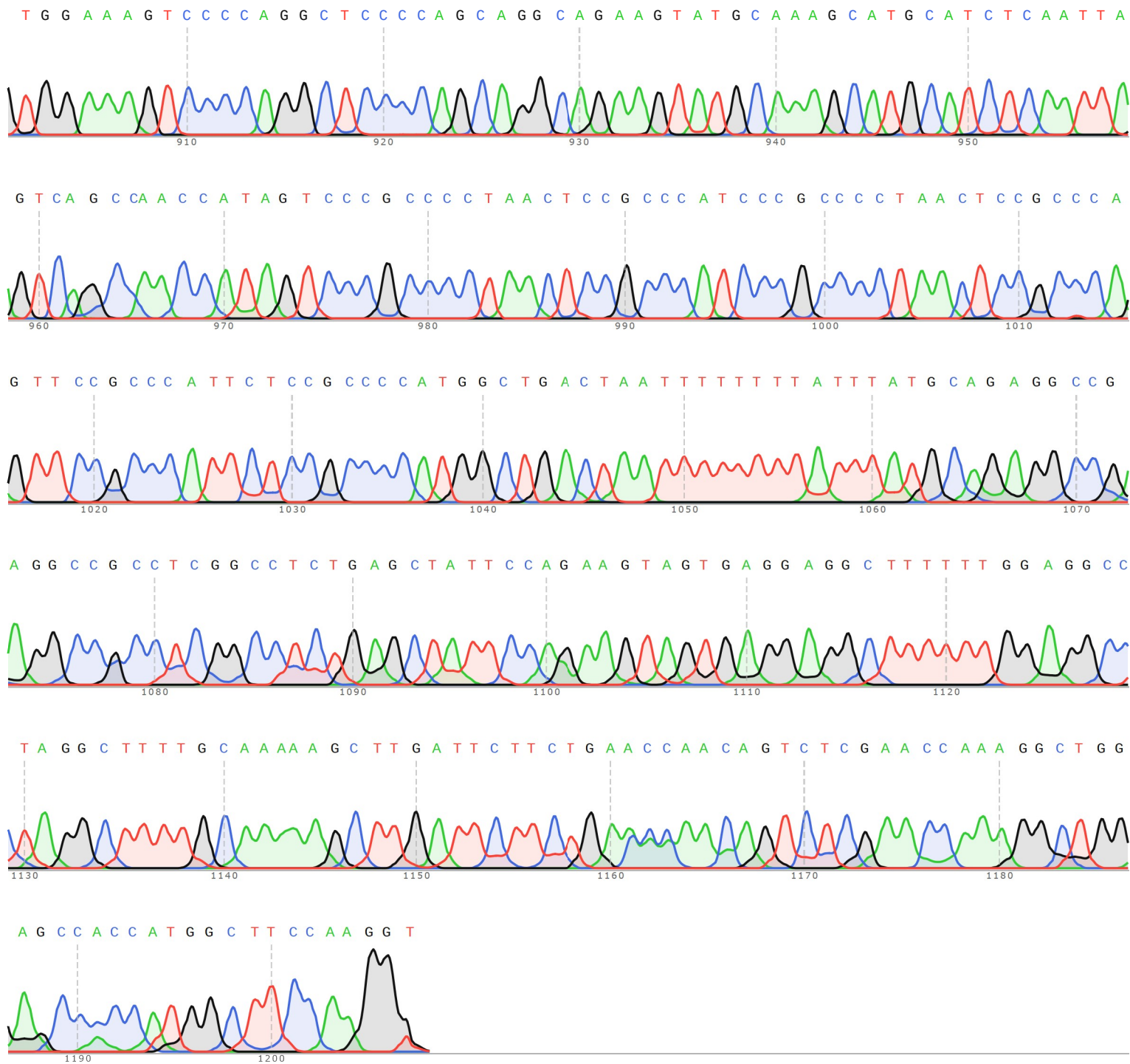

Supplement: Supplementary file 1 [file vetsci-12-00095-s001.zip › vetsci-3307142-supplementary/supplementary file/supplementary figures/Figure S3-RAB37 mutant.pdf]

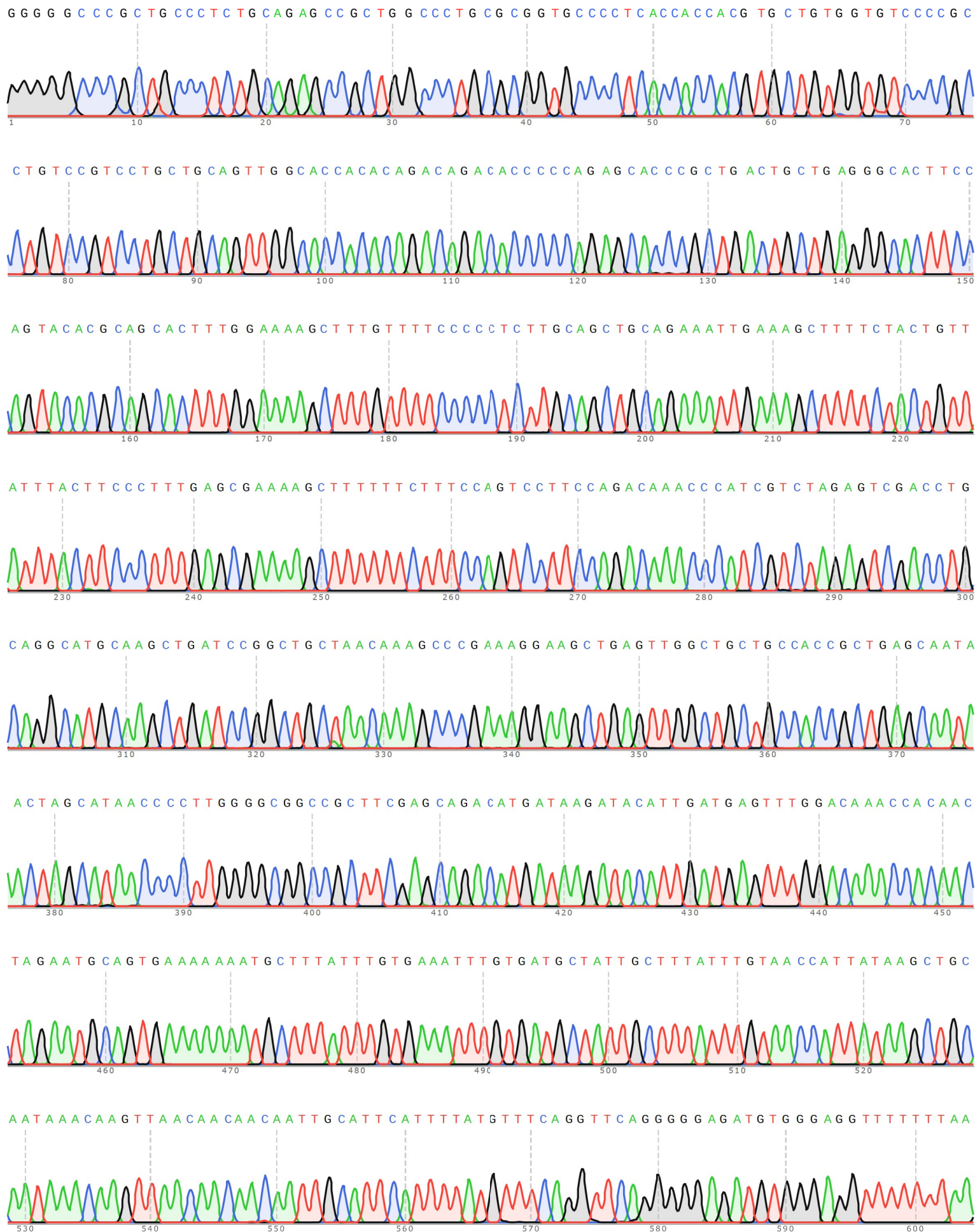

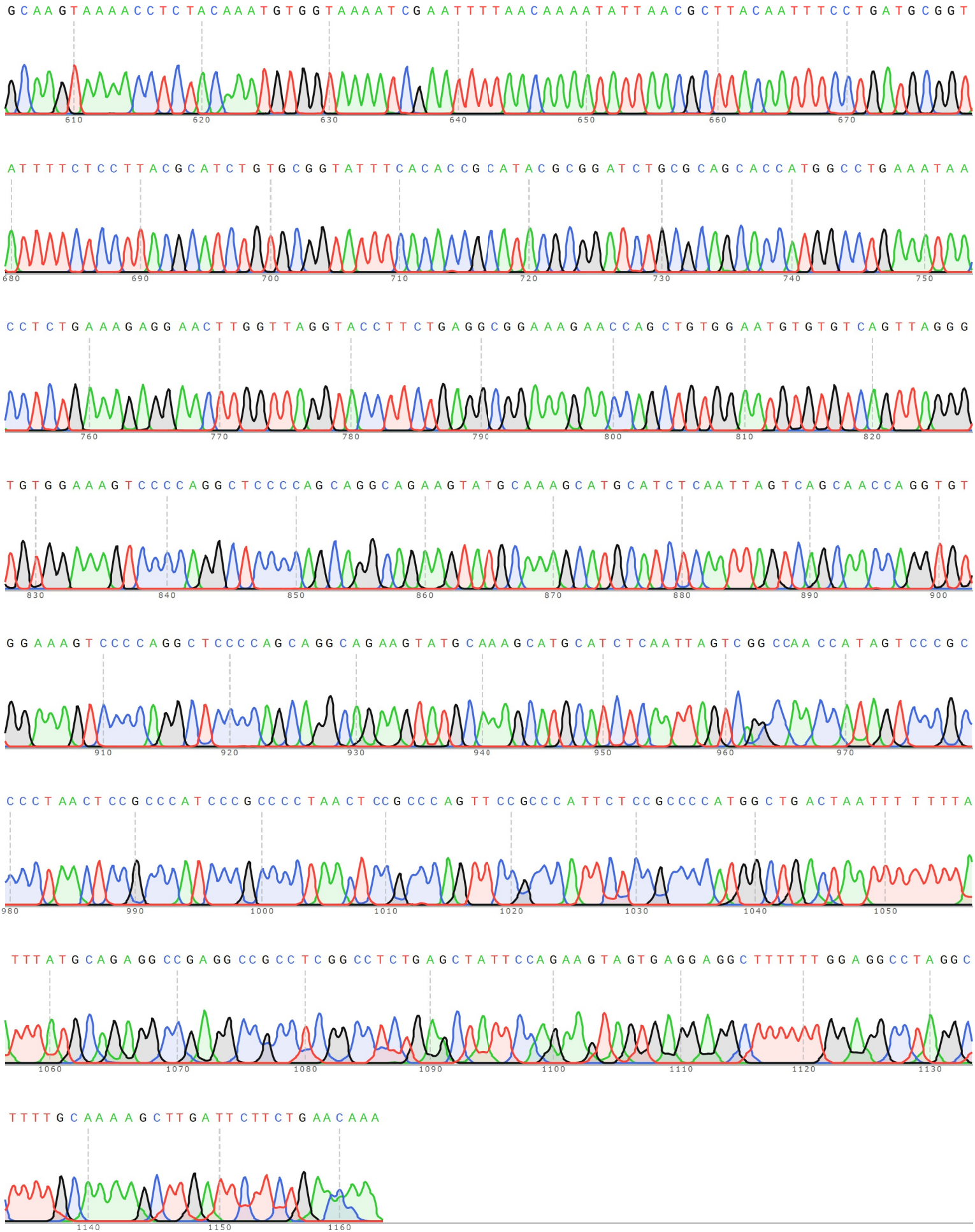

Supplement: Supplementary file 1 [file vetsci-12-00095-s001.zip › vetsci-3307142-supplementary/supplementary file/supplementary figures/Figure S4-RAB37 wild type.pdf]
